# Resistance to Vemurafenib Can Be Reversible After Treatment Interruption: A Case Report of a Metastatic Melanoma Patient

**DOI:** 10.1097/MD.0000000000000157

**Published:** 2014-12-12

**Authors:** Małgorzata Mackiewicz-Wysocka, Łukasz Krokowicz, Jacek Kocur, Jacek Mackiewicz

**Affiliations:** From the Department of Dermatology (MM-W), Poznan University of Medical Sciences; Department of General, Endocrinological and Gastroenterological Oncology Surgery (LK), Poznan University of Medical Sciences; Department of Radiology (JK), Greater Poland Cancer Centre; Department of Medical Biotechnology (JM), University of Medical Sciences; Department of Diagnostics and Cancer Immunology (JM), Greater Poland Cancer Centre, Poznan; and Department of Medical Oncology (JM), Małgorzata Medical Center, Srem, Poland.

## Abstract

About 40% to 60% of melanomas present *BRAF* mutation. Selective BRAF inhibitors such as vemurafenib and dabrafenib are currently approved for the treatment of advanced melanoma patients with *BRAF* mutation. The treatment-induced tumor regression occurs in the majority of patients; however, acquired resistance to BRAF inhibitors is observed in most of the patients after 6 to 7 months. After progression of the disease, the patient might be offered treatment with ipilimumab followed by chemotherapy. Subsequent lines of systemic treatment of metastatic melanoma patients do not exist.

Here we report a case of a 59-year-old woman with a diagnosis of *BRAF*-mutant metastatic melanoma that responded to initial treatment with vemurafenib. Subsequently, after disease progression, the patient received chemotherapy. Since no clinical response to dacarbazine was observed, carboplatin with paclitaxel were applied. Transient partial response was obtained, which was followed by further disease progression. Then retreatment with vemurafenib was applied. The patient developed very short-term tumor regression and significant biochemical response (serum lactate dehydrogenase, alanine aminotransferase, and aspartate aminotransferase) to the treatment. However, following 5 weeks of retreatment, the patient developed progression of the disease. Our clinical observation indicates that in melanoma patients who developed resistance to selective BRAF inhibitors, rechallenge after treatment interruption might be beneficial.

## INTRODUCTION

Forty percent to 60% of melanomas harbor a driver mutation, mainly *V600E* or *V600K,* in *BRAF* gene. Randomized trials have shown that metastatic melanoma patients with *BRAF* mutation benefit from treatment with selective BRAF inhibitors—vemurafenib (Zelboraf; Roche, Basel, Switzerland) and dabrafenib (Tafinlar; GlaxoSmithKline, Research Triangle Park, NC). About 80% of patients with *BRAF*-mutated metastatic melanoma treated with BRAF inhibitors display tumor regression with a partial response in approximately 50% of patients. However, most tumors develop resistance to the treatment within 6 to 7 months.^1,2^ Currently, the number of possible mechanisms of resistance has been described. They were mainly related to the reactivation of extracellular signal-regulated kinases, the downstream effectors in the mitogen-activated protein kinases pathway.^3,4^

Standard treatment with vemurafenib consists of daily oral administration of the drug until progression of the disease. In the second-line treatment, patients might be offered treatment with ipilimumab followed by chemotherapy. Subsequent lines of systemic treatment outside the clinical trials do not exist and metastatic melanoma patients with good performance status are left without treatment.

To our knowledge, only 2 studies have reported rechallenge of vemurafenib treatment in 3 patients after initial administration of this drug.^5,6^

We report a case of a *BRAF*-mutant metastatic melanoma patient who responded to initial treatment with vemurafenib. Subsequently, after therapy failure, the patient received 2 lines of chemotherapy and further disease progression was re-treated with vemurafenib.

## CASE REPORT

The patient was a 59-year-old woman who in December 2009 had resected primary skin melanoma demonstrating characteristics of regression (pTis). In parallel, excision of 2 in-transit metastases from the left lower limb was performed. Subsequently, another 2 in-transit metastases were resected in August and September 2010, respectively. In September 2011, the patient developed metastases to the lungs and subcutaneous tissue in the left lower limb. In October 2011, the patient was enrolled to Expanded Access Program (MO25515) evaluating vemurafenib in patients with advanced *BRAF*-mutant melanoma. At that time, the computed tomography (CT) scan demonstrated additional metastases to the mediastinal and iliac lymph nodes. After 9 months of vemurafenib treatment, the patient continued stabilization of the disease with shrinkage of metastatic lesions. In September 2012, one nontarget lesion appeared with stabilization of other monitored lesions. The patient continued treatment with vemurafenib. In November 2012, she developed metastases in lungs, liver, lymph nodes, and subcutaneous tissue, thus the treatment with vemurafenib was discontinued.

Because of lack of reimbursement of ipilimumab (Yervoy; Bristol-Myers Squibb, New York City, NY) therapy in Poland, the patient was applied the second-line treatment with dacarbazine (Dacarbazin TEVA; TEVA, Petah Tikva, Israel) (200 mg/m^2^ day 1–5 every 3 weeks). After 3 cycles of chemotherapy, progression of the disease was observed. Patient's performance status was very good (Eastern Cooperative Oncology Group [ECOG] 0) and she did not demonstrate any toxicity related with chemotherapy. In February 2013, the patient received third line of systemic treatment composed of carboplatin (Paraplatin; Bristol-Myers Squibb) (AUC 6) and paclitaxel (Taxol, Bristol-Myers Squibb) (175 mg/m^2^) administered every 3 weeks. In June 2013, the patient demonstrated partial response after 6 cycles of carboplatin and paclitaxel. Because of hematological toxicity and poor tolerance of chemotherapy, the treatment was interrupted and the patient was followed for 3 months without any anticancer treatment. In September 2013, the patient developed massive progression in the lungs, mediastinal lymph nodes, liver, spleen, kidney, and bones.

Because of the lack of effective therapeutic options in this indication, we decided to meet the patient's expectations and retreatment with vemurafenib as standard dose in September 2013. Patient was in a good performance status with ECOG 0. Before starting the treatment, lactate dehydrogenase (LDH) serum level was 2930 U/L (laboratory norm: 135–225 U/L) and the alanine aminotransferase (ALT) and aspartate aminotransferase (AST) both were elevated (grade 1). After 1 week of treatment, the level of LDH decreased to 740 U/L. Aminotransferases have also decreased but were still grade 1. After 2 weeks of treatment, LDH level was 606 U/L and the aminotransferases were in normal reference range. At that time, shrinkage of the lesions in the subcutaneous tissue in the left lower limb was observed. However, after 3 weeks from the start of vemurafenib therapy, LDH level started to increase up to 770 U/L and continued up to 1061 and 1209 U/L after 4 and 5 weeks of treatment, respectively. Moreover, enlargement of subcutaneous metastases in the left lower limb was observed. The CT scan performed following 4 to 5 weeks of retreatment with vemurafenib demonstrated progression of the disease (enlargement of the majority of earlier observed lesions with the occurrence of new metastases). Patient's performance status worsened with ECOG 2. Treatment with vemurafenib was interrupted and after 2 weeks the patient died.

## DISCUSSION

Recently, Romano et al^5^ reported a case of a patient with *BRAF*-mutant metastatic melanoma retreated with vemurafenib. Earlier the patient received vemurafenib in the first-line setting. After disease progression, the patient received 4 cycles of ipilimumab followed by 2 cycles of temozolomide (Temodal, Merck Sharp & Dohme, Whitehouse Station, NJ) and 1 cycle of fotemustine (Muphoran, Servier Laboratories, Neuilly-sur-Seine, France). Subsequently, after chemotherapy failure, the patient was rechallenged with vemurafenib (10 months from initial administration of vemurafenib) developing a partial response lasting 4 months.^5^

In other case report, Seghers et al^6^ demonstrated 2 patients with *BRAF*-mutant metastatic melanoma who progressed after treatment with dabrafenib/trametinib and dabrafenib, respectively. After 8 and 4 months of interval, these patients were rechallenged with dabrafenib and vemurafenib, respectively, developing mixed and partial responses.^6^

In our patient, we observed a rapid decline (after 1 week) of LDH and regression of skin metastases (after 2 weeks) after retreatment with vemurafenib. The rapid LDH decline indicates that the patients previously responded to therapy^7^; however, the response to the treatment was transitional and the patient benefit too was questionable. Although the patient did not demonstrate any adverse events during vemurafenib rechallenge.

The above described cases and our experience indicate that the resistance to BRAF inhibitors might be temporary. The mechanisms of the phenomenon might be due to epigenetic changes or specific microenvironment conditions.^6^ It is also possible that in our case, chemotherapy with carboplatin and paclitaxel eliminated partially BRAF-resistant clones, while those sensitive to vemurafenib survived and responded to the retreatment. On the contrary, Das Thakur et al^8^ demonstrated in a human melanoma xenograft model that intermittent vemurafenib dosing forestalls the development of drug resistance showing that durability of responses to BRAF inhibitors may be improved through alterations in the dosing schedule.^8^ Reversible drug tolerance has been observed in non-small cell lung cancer (NSCLC) cells retreated with tyrosine kinase inhibitors (TKIs).^9^ Moreover, the so-called “retreatment response” has been noted in patients with advanced NSCLC rechallenged with TKIs.^10,11^ Nevertheless, these findings suggest that such acquired resistance to small molecule drugs may involve a reversible drug-tolerant state whose mechanism remains somewhat unclear.

These observations show that resistance to BRAF inhibitors can be reversible after treatment interruption. However, retreatment with BRAF inhibitors in *BRAF*-mutant metastatic melanoma patients after earlier effective therapy with vemurafenib/dabrafenib might be a treatment option but not for all patients. It might be very difficult to define the proper patient population that might benefit from BRAF inhibitors rechallenge due to heterogeneity of melanoma cells after previous treatment with these drugs. However, these findings are encouraging and need exploration in the near future.
